# A fully automated flow injection atomic absorption system for the determination of copper traces in waters with on-line pre-concentration in an ion-exchange column

**DOI:** 10.1155/S1463924695000046

**Published:** 1995

**Authors:** J. L. Burguera, M. Burguera, P. Carrero, J. Marcano, C. Rivas, M. R. Brunetto

**Affiliations:** Department of Chemistry Faculty of Sciences University of Los Andes PO Box 542 Mérida 5101-A Venezuela

## Abstract

The paper describes the development of an automatic on-line column pre-concentration technique using a time based-flow injection atomic absorption spectrometry system. A manifold incorporating a micro-column containing 25 mg of Dowex 50W-X8 was used with a time-based injector for the pre-concentration and determination of copper in natural and drinking waters. The system features depend on the alternate positions of a solenoid valve. The 3σ detection limits, enrichment factors, sampling frequency, relative standard deviations and linear calibration graphs were, respectively, in the range 0.6-1.5 μg/l, 25-60, 15-30 measurements/h, 1.0-3.1% and 1-65 μg/ml for pre-concentration times of 1 min. The procedure was successfully applied to a range of water samples and the accuracy was assessed through recovery experiments, the analysis of certified reference water samples and by independent analysis by atomic absorption spectrometry with electrothermal atomization.


Journal of Automatic Chemistry, Vol. 17, No. 1 (January-February 1995), pp. 25-29

### A fully automated flow injection atomic absorption system for the determination of copper traces in waters with on-line pre-concentration in an ion-exchange column

**J. L. Burguera, M. Burguera, P. Carrero, J. Marcano, C. Rivas and M. R. Brunetto**

*Department of Chemistry, Faculty of Sciences, University of Los Andes, PO Box 542, Mérida 5101-A, Venezuela*

*The paper describes the development of an automatic on-line column pre-concentration technique using a time based-flow injection atomic absorption spectrometry system. A manifold incorporating a micro-column containing 25 mg of Dowex 50W-X8 was used with a time-based injector for the pre-concentration and determination of copper in natural and drinking waters. The system features depend on the alternate positions of a solenoid valve. The 3σ detection limits, enrichment factors, sampling frequency, relative standard deviations and linear calibration graphs were, respectively, in the range 0·6–1·5 μg/l, 25–60, 15–30 measurements/h, 1·0–3·1% and 1–65 μg/ml for pre-concentration times of 1 min. The procedure was successfully applied to a range of water samples and the accuracy was assessed through recovery experiments, the analysis of certified reference water samples and by independent analysis by atomic absorption spectrometry with electrothermal atomization.*

### Introduction

Ion exchangers used in miniature columns have proven useful for both analyte concentration and the removal of interfering compounds. However, the conventional column mode of ion-exchange pre-concentration is both tedious and time consuming compared with the final rapid determination of atomic spectroscopy (AS) techniques [1]. The use of ion exchange as an auxiliary technique with flow injection (FI)-AS results in on-line increased sensitivity with respect to the direct aspiration procedure, speeding up and simplying the pre-concentration step and the implementation of simultaneous determinations and/or speciation [2].

Manually and electronically operated injection and pneumatic valves and commutators have been employed for sample introduction in ion exchangers—FI-AAS manifolds [3, 4]. These introduction devices permit the reproducible and sequential introduction of defined sample volumes into flowing systems, but they are wasteful in terms of the sample. In order to remove the residue of the previous specimen, an excess of sample must be drawn through the valve and connecting tubes. This paper reports on an attempt to develop a fully automated on-line system with an electronically operated time-based injector for high efficiency, low sample and reagents consumption ion-exchange pre-concentration. The FI-AAS system described was designed for the determination of copper at the sub-μg/ml level in water.

### Materials and methods

### *Apparatus*

A schematic diagram of the flow system is given in figure 1. AAS measurements were performed with an air–acetylene flame on a Varian AA-1475 atomic absorption spectrometer interfaced to a Varian 9176 strip chart recorder. The suction air-pressurized device consisted of a bottle with a pressure regulator up to 40 cm Hg and an Iwaki AP-115 pump which produces the suction pressure. A Gilson Minipuls 2 peristaltic pump and poly(tetrafluoroethylene) (PTFE) tubing of 0·8 mm i.d. was used throughout. The conical extraction pre-concentration column was made from a 200 μl plastic Eppendorf pipette tip packed with 25 mg of Dowex 50W-X8 (100–200 mesh) ion exchanger. Tips were cut to provide an outlet of approximately 0·8 mm i.d. and the exchanger was kept in place with small glass-wool beds at each end of the tip. Connections to the column were made using a combination of short tubes with appropriate bore diameters slipped either inside (on the wider side) or outside (on the narrower side) the column.

The time-based injector [5] consisted of a Gralab 900 timer which sends electronically controlled pulses to a solenoid valve. The solenoid valve can be activated to close or open (by pressing or releasing) any selected set of PTFE tubing at fixed times [5, 6], thus allowing sequestration of sample and/or eluent.

For accuracy experiments, electrothermal atomic absorption spectrometry (ETAAS) with deuterium background correction (Perkin-Elmer Model 2100 equipped with an HGA-700 furnace and an AS-90 furnace autosampler) was used; Perkin-Elmer pyrolitic graphite coated tubes and platforms were also used.

### *Reagents and samples*

Reagents of analytical-reagent grade (E. Merck, Darmstadt, Germany) and de-ionized water (18 MΩ/cm) prepared with a Millipore water-purification system were used.

0142-0453/95 $10.00 © 1995 Taylor & Francis Ltd.

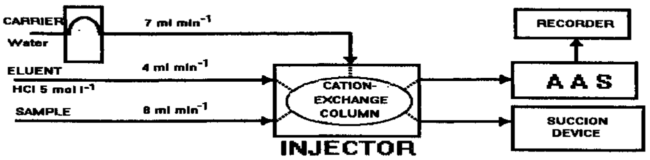

*Figure 1. Schematic diagram of the flow system.*

A stock standard solution of Cu (1000 mg/l) was prepared in 0·1 N nitric acid. The water samples were analysed on the collection date without pre-treatment. All samples and working standards were acidified to 0·1 N with nitric acid.

Reference materials were obtained from the National Institute of Standards and Technology (NIST) and Promochem (Promochem, Wesel, Germany). Water samples from the Mucujun River (a tributary of the Chama River [7]) and domestic water sources from the city of Mérida were analysed.

Dowex 50W-X8 (100–200 mesh) (BDH) resin was used for preparing the minicolumn.

*Procedure*

The set-up and operating principle of the flow system is shown in figure 2. In step 1 (loading position: figure 2(*a*)) the carrier is pumped for 60 s directly to the spectrometer, while the eluent and sample (through the column) solutions were drawn to waste. The manifold was arranged in such a way that the flow of the sample enters through the narrower end of the column and the bulk of the absorbed analyte is retained on this side to minimize dispersion. In step 2 (elution position; figure 2(*b*)) the solenoid valve is switched to the alternate position for 60 s and in this way the eluent is directed from the broader to the narrower end of the column. Therefore, the analyte was eluted in the counter direction and directed to the nebulizer of the spectrometer. All the results were based on peak height (absorbance measurements).

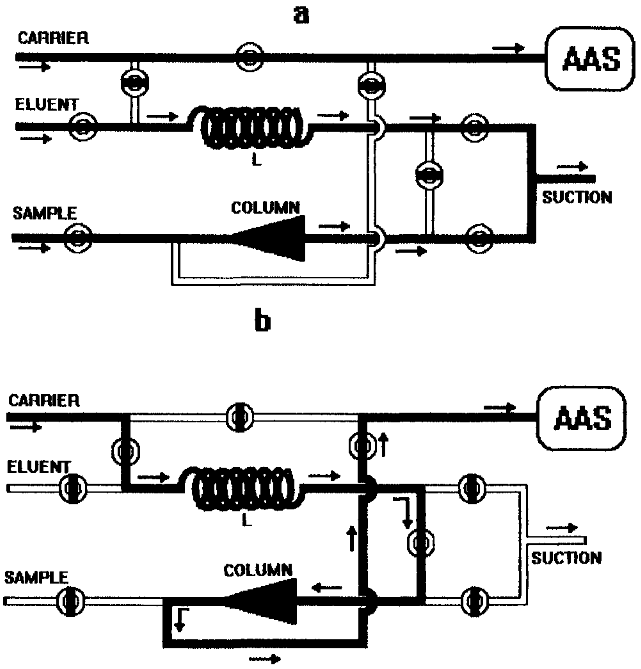

*Figure 2. The set-up and operating principle of the flow system. (a) Loading and (b) elution positions. (◉) and (◎) indicate closed and open channels, respectively.*

### Results and discussion

*Operating conditions*

The one-factor-at-a-time method was used to optimize the experimental parameters, while the rest were kept constant. The operating conditions chosen were those which produced the highest and most reproducible signals (table 1).

*Table 1. Operating conditions for the on-line pre-concentration of copper in the time-based FI-AAS system.*

| System | Parameter | Conditions |
|---|---|---|
| Flame AAS | Wavelength (nm) | 324·6 |
| | Lamp current (mA) | 3 |
| | Slit width (nm) | 0·5 |
| | Air and acetylene flow rates (l/min) | 21·0 and 2·0 |
| Chemical | HCl eluent concentration (mol/l) | 5 |
| Flow injection | Sample flow rate (ml/min) | 8 |
| | Eluent flow rate in the loading position (ml/min) | 4 |
| | Elution flow rate (ml/min) | 7 |
| | Sample volume (ml) | 8 |
| Injector | Time of loading (min) | 1 |
| | Time of elution (min) | 1 |
| | Suction pressure (cm Hg) | 45 |
| Microcolumn | Column size | 0·2 ml tip |
| | Resin | Dowex 50W-X8 (100–200 mesh) |
| | Resin amount | 25 mg |

*Table 2. Influence of sample volume on the performance of the on-line ion-exchange pre-concentration AAS system using a Dowex 50W-X8 resin.*

| Time of sample loading (min) | Sample volume (ml) | Equation* | Linear range (ng/ml) | Detection limit† (ng/ml) | Enrichment factor‡ (EF) |
|---|---|---|---|---|---|
| 1 | 8 | A = 0·002 + 0·002 [Cu] | 2–65 | 1·5 | 25 |
| 2 | 16 | A = 0·003 + 0·004 [Cu] | 1–45 | 0·9 | 41 |
| 3 | 24 | A = 0·004 + 0·005 [Cu] | 1–30 | 0·6 | 60 |

* A = absorbance and [Cu] = copper concentration in ng/ml.
† Considered as the amount of copper which gives an absorbance value corresponding to twice the standard deviation of the blank signal.
‡ Expressed as the ratio of the slopes of the respective calibration graphs of eluted analyte from aqueous standards and the same solution using conventional sample introduction.

*Influence of sample volume*

In the configuration of the time-based injector used in this work, almost any desirable sample volume can be introduced by increasing the solenoid activation time and the pressure of aspiration or suction of sample and eluent. However, the sample volume cannot be increased at will. First, at higher injected volumes there is a drop in reproducibility (table 2); for example the precision deteriorated as the relative standard deviation (RSD) increased from 2·3 to 3·2% for the introduction of 8 to 24 ml (solenoid activation times of 1 and 3 min, respectively) of sample volume. Second, if the solenoid of the injector sticks in the open position for more than 2 min, the coil through which current is flowing is likely to burn out—during the optimization of the sample volume parameter the solenoid unit was replaced four times. As the introduction of different sample volumes and the fixation of a given desired volume in function of pressure of aspiration varies considerably from one day to another, it was decided to vary the solenoid activation time in order to fix a given volume.

The analytical characteristics of the system depend on the sample volume introduced (table 2). At higher sample volumes, the detection limit was lower but the enrichment factor (concentration efficiency) and the sensitivity of measurements increased; however, the linear working range and the sampling frequency were reduced. A sample volume of 8 ml seems to be a good compromise between good sensitivity, sampling frequency, concentration efficiency and time of activation of the solenoid.

*Effect of the amount of ion-exchanger*

When the column was packed with increased amounts of resin, only slight decreases in the retention capacity of copper was observed. This effect could be due to the fact that conical shaped columns are less sensible to dispersion processes [8] than uniform diameter columns, if the elution step is performed in the counter direction to that of the loading step. In addition, as the aspiration rate in the loading position is determined by the capability of the pump suction, an increase in the back-pressure and flow rate instabilities were observed with larger amounts of resin. Low enrichment factors (< 15) were obtained when small amounts of resin (< 10 mg) were used due to saturation of the adsorption sites by the analyte. Taking these factors into account, 25 mg of ion-exchanger offers the best conditions for both the deposition and elution of copper and was therefore chosen for this study.

*Optimization of hydrodynamic and chemical conditions*

The deposition flow rate for the copper on the ion exchange resin was varied between 1 and 12 ml/min, while the elution flow rate was held constant at 7 ml/min. Optimization studies have shown that an increase in sample-loading rate enhances the absorbance measurements within a certain flow-rate interval (from 1 to 8 ml/min). At flow rates above 8 ml/min, the signal can be seen to decrease (figure 3) and this is due in part to the column dimensions, the capacity of the ion-exchanger and to increased dispersion of analyte in the dead volume of the column.

The peristaltic pump was adjusted to give a delivery rate of 7 ml/min for the carrier solution which displaces the eluting zone contained in the loop L of figure 2(*a*), The

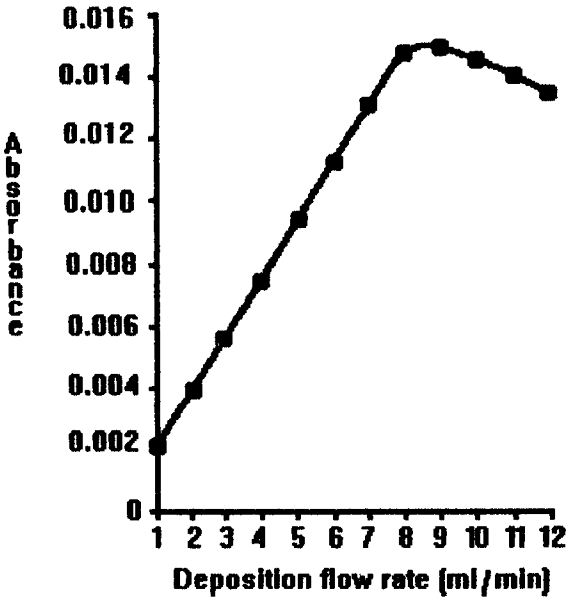

*Figure 3. Deposition flow rates as a function of signal response for a 6·5 ng/ml of copper.*

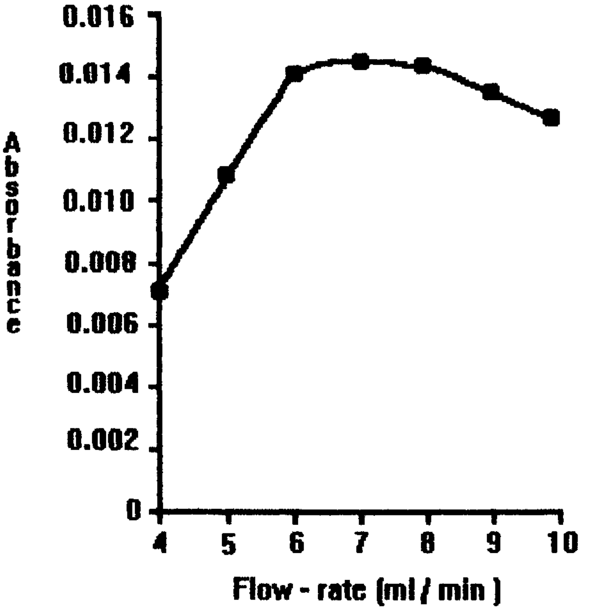

*Figure 4. Effect of carrier-eluent flow rate on the peak height (absorbance); ng/ml solution of copper.*

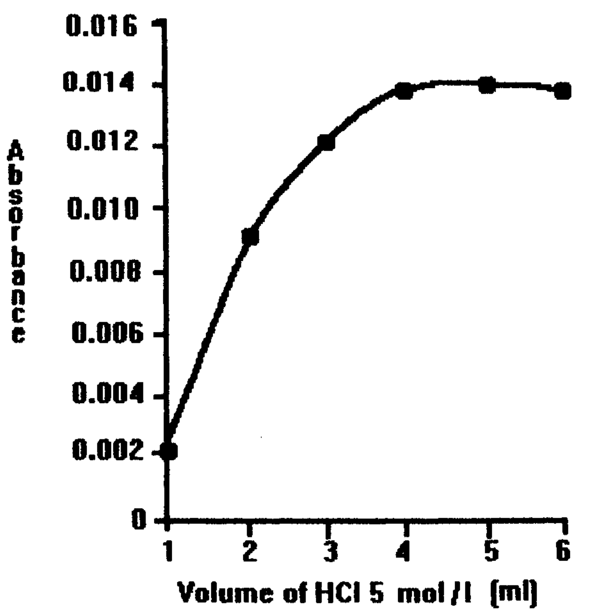

*Figure 5. Signal response for 6 ng/ml of copper with variation of eluent volume.*

results (figure 4) indicated that the analyte elution was similar in the eluent flow rate range 6·5–8 ml/min. At flow rates above and below this range the signal can be seen to decrease. Faster flow rates increased dispersion and decreased the absorbance.

The use of elution volumes below 4·0 ml proved to be insufficient to recover the resin-retained copper (figure 5). The absorbance signal was virtually unchanged at elution volumes above 4·0 ml. Besides, this eluent volume, which is directly related to the elution flow rate and the acid concentration, served to remove any analyte that may be present in the column and in the lines. Also, it was sufficient for column regneration between each sample injection.

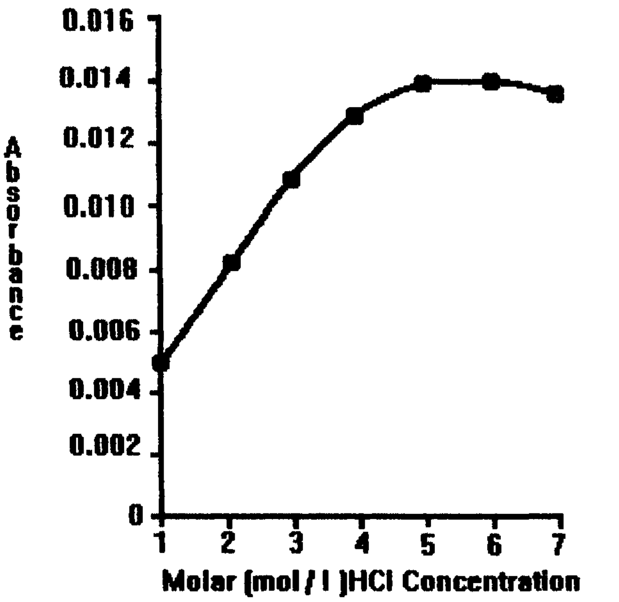

*Figure 6. Effect of eluent concentration on the magnitude of the signal for 6 ng/ml solution of copper.*

Figure 6 is a plot of the signal for the copper eluted versus concentration of HCl. As expected, the results indicate an increase in signal intensity as the acid concentration increases from 1 to 5 mol/l. It was found that an increase in the acid concentration above 4·5 mol/l did not enhance the signal. Therefore, this acid concentration is advised as higher concentrations may affect the column material and deteriorates the FI tubing.

*Matrix effect studies*

In order to study the effect of the matrix on absorbance, standard addition graphs were prepared by adding various amounts of copper (from 2 to 10 mg $ml^{-1}$) to river and tap water samples. The final concentration of the element was never allowed to be outside its linear range under the analytical conditions used here. As the slopes of standard additions graphs were not significantly different (difference between slopes $<3\%$) from those obtained with copper standard, it was concluded that there is not an appreciable effect of the matrix components on the copper determination.

*Figures of merit*

The linear ranges, detection limits, sampling frequencies and enrichment factors listed in table 3 for different sample volumes were obtained under the conditions outlined in table 1.

The precision of the method was checked by calculating the RSD of eight sets of replica determinations on 14 samples containing from 4·2 to 18·8 ng/ml of copper. The RSD was in the range 0·6–2·5% for the pre-concentration of 8, 16 and 24 ml of sample.

The accuracy of the method was investigated by determining the copper content of two standard reference materials (SRM) (table 3), in water samples collected from seven different sampling sites of the Mucujun River and in the

*Table 3. Copper content found in standard matrials using the proposed method*.*

| Material | Certified copper content | Copper content found |
|---|---|---|
| NIST SRM 1643b trace elements in water | 22·3 ± 0·4 | 22·5 ± 0·5 |
| Promochem SLR S2 river water | 2·76 ± 0·03 | 2·81 ± 0·06 |

* Values in ng/ml.

*Table 4. Determination of copper in tap and river waters by time-based FI-FAAS with ion-exchange pre-concentration and ETAAS*.*

| Water | Sample | FI-FAAS | ETAAS |
|---|---|---|---|
| Tap | 1 | 4·2 ± 0·1 | 4·6 ± 0·3 |
| | 2 | 5·3 ± 0·2 | 5·1 ± 0·3 |
| | 3 | 4·6 ± 0·1 | 4·7 ± 0·2 |
| | 4 | 6·2 ± 0·1 | 5·9 ± 0·1 |
| | 5 | 3·1 ± 0·3 | 2·8 ± 0·3 |
| | 6 | 5·1 ± 0·2 | 5·3 ± 0·2 |
| | 7 | 6·0 ± 0·1 | 6·3 ± 0·2 |
| River | 1 | 8·5 ± 0·5 | 8·2 ± 0·3 |
| | 2 | 13·5 ± 0·3 | 13·8 ± 0·4 |
| | 3 | 10·3 ± 0·1 | 9·9 ± 0·2 |
| | 4 | 13·0 ± 0·1 | 13·1 ± 0·4 |
| | 5 | 18·5 ± 0·3 | 19·0 ± 0·4 |
| | 6 | 11·2 ± 0·2 | 11·0 ± 0·3 |
| | 7 | 14·1 ± 0·1 | 13·9 ± 0·3 |

* Mean of three determinations ± standard deviation in ng/ml.

tap water of Mérida City [9] (table 4). In addition, accuracy was assessed with recovery experiments. Recovery of copper was checked by the addition of µl-aliquots of copper standard solution to tap and river waters; the recoveries of copper (2–5 ng/ml) were between 95 and 102%. These results show good agreement with results obtained with the methods in routine use in the authors' laboratory, which is an indication that the accuracy of the proposed method is satisfactory.

### Conclusions

Connecting a time-based on-line pre-concentration system to AAS has been shown to increase the speed of the pre-concentration analysis process and reduces sample manipulation and consumption. The sampling frequency was 30 measurements/h with a sample and eluent consumption rate of 8 and 7 ml per determination, respectively. An exciting aspect of this approach is that the sample and reagents are not consumed during the stoppage of the sample and eluent, thus their dead volumes are practically eliminated. The system should be useful for other applications incorporating ion exchangers with colorimetry, potentiometry and AS.

### Acknowledgements

This study was supported by the CONICIT (Consejo Nacional de Investigaciones Científicas y Tecnológicas) project S1 and the CDCHT (Consejo de Desarrollo Científico y Humanístico) of the Andes University.

### References

1. Stewart, K. K., *General Introduction, in Flow Injection Atomic Spectroscopy*, Ed. Burguera, J. L. (M. Dekker, New York, 1989).
2. Knapp, G., Muller, M., Strunz, M. and Wegscheider, W., *Journal of Analytical Atomic Spectrometry*, **3** (1987), 611.
3. Zagatto, E. A. G., Krug, F. J., Bergamin, H. and Jorgensen, S. S., *Flow Injection Atomic Spectroscopy* (M. Dekker, New York, 1989).
4. Karlberg, B. and Pacey, G. E., *Flow Injection Analysis. A practical Guide* (Elsevier, Amsterdam, 1989).
5. Carrero, P., Burguera, J. L., Burguera, M. and Rivas, C., *Talanta*, **40** (1993), 1967.
6. Burguera, J. L., Burguera, M. and Brunetto, M. R., *Atomic Spectroscopy*, **14** (1993), 90.
7. Burguera, J. L., Burguera, M., Rivas, P. C., Fontana de Wilhelm, L. and Oropeza, B. J. G., *Acta Cientifica Venezoilana*, **37** (1986), 657.
8. Fang, Z. and Welz, B., *Journal of Analytical Atomic Spectrometry*, **4** (1989), 543.
9. Perkin-Elmer Corporation, *Techniques in Graphite Furnace Atomic Absorption Spectrometry*, Part No. 0993-8150 (Perkin-Elmer Corp., Ridgefield, CT, USA, 1985).

